# Articular cartilage generation applying PEG-LA-DM/PEGDM copolymer hydrogels

**DOI:** 10.1186/s12891-016-1100-1

**Published:** 2016-06-03

**Authors:** Xing Zhao, Anestis Papadopoulos, Shinichi Ibusuki, David A. Bichara, Daniel B. Saris, Jos Malda, Kristi S. Anseth, Thomas J. Gill, Mark A. Randolph

**Affiliations:** Department of Orthopaedic Surgery, Massachusetts General Hospital, Harvard Medical School, Boston, MA USA; Division of Plastic Surgery, Massachusetts General Hospital, Harvard Medical School, WACC 435, 15 Parkman Street, Boston, MA 02114 USA; Department of Orthopaedics, University Medical Center Utrecht, Utrecht, The Netherlands; MIRA Institute for Biotechnology and Technical Medicine, University Twente, Enschede, The Netherlands; Department of Equine Science, Faculty of Veterinary Medicine, Utrecht University, Utrecht, The Netherlands; Department of Chemical and Biological Engineering, University of Colorado, Boulder, CO USA

**Keywords:** Cartilage regeneration, Articular cartilage, PEG hydrogel, PEGDM hydrogel

## Abstract

**Background:**

Injuries to the human native cartilage tissue are particularly problematic because cartilage has little to no ability to heal or regenerate itself. Employing a tissue engineering strategy that combines suitable cell sources and biomimetic hydrogels could be a promising alternative to achieve cartilage regeneration. However, the weak mechanical properties may be the major drawback to use fully degradable hydrogels. Besides, most of the fully degradable hydrogels degrade too fast to permit enough extracellular matrix (ECM) production for neocartilage formation. In this study, we demonstrated the feasibility of neocartilage regeneration using swine articular chondrocytes photoencapsualted into poly (ethylene glycol) dimethacrylate (PEGDM) copolymer hydrogels composed of different degradation profiles: degradable (PEG-LA-DM) and nondegradable (PEGDM) macromers in molar ratios of 50/50, 60/40, 70/30, 80/20, and 90/10.

**Methods:**

Articular chondrocytes were isolated enzymatically from juvenile Yorkshire swine cartilage. 6 × 10^7^ cells cells were added to each milliliter of macromer/photoinitiator (I2959) solution. Nonpolymerized gel containing the cells (100 μL) was placed in cylindrical molds (4.5 mm diameter × 6.5 mm in height). The macromer/photoinitiator/chondrocyte solutions were polymerized using ultraviolet (365 nm) light at 10 mW/cm^2^ for 10 mins. Also, an articular cartilaginous ring model was used to examine the capacity of the engineered cartilage to integrate with native cartilage. Samples in the pilot study were collected at 6 weeks. Samples in the long-term experimental groups (60/40 and 70/30) were implanted into nude mice subcutaneously and harvested at 6, 12 and 18 weeks. Additionally, cylindrical constructs that were not implanted used as time zero controls. All of the harvested specimens were examined grossly and analyzed histologically and biochemically.

**Results:**

Histologically, the neocartilage formed in the photochemically crosslinked gels resembled native articular cartilage with chondrocytes in lacunae and surrounded by new ECM. Increases in total DNA, glycosaminoglycan, and hydroxyproline were observed over the time periods studied. The neocartilage integrated with existing native cartilage.

**Conclusions:**

Articular cartilage generation was achieved using swine articular chondrocytes photoencapsulated in copolymer PEGDM hydrogels, and the neocartilage tissue had the ability to integrate with existing adjacent native cartilage.

## Background

Lesions in the knee joint surface are commonly treated with microfracture [1], autologous cell implantation (ACI) [2], or osteoarticular autograft transfer system (OATS) [3]. Although patients have symptomatic relief, there is little convincing histological or biochemical data to support the contention that the new tissue that forms is characteristic of native hyaline cartilage found on the joint surface that is composed predominantly of type II collagen. ACI and microfracture most often result a fibrous cartilage repair that is high in type I collagen and not durable in weight bearing positions over the long term. Roberts et al. have reported that as many as 65 % of second look biopsies showed fibrocartilage [4]. Recent publications including a meta-analysis suggest there are no differences among ACI, microfracture, and OATS [5, 6]. Newer modifications of the ACI technique being tested in Europe involve an open weave or sponge matrix (MACI), frequently made from collagen or hyaluronic acid, where the cells are absorbed into the matrix before being secured in the lesion [7]. These woven type scaffolds do not provide any immediate biomechanical integrity and can be crushed by the forces placed on the joint. Furthermore, recent results suggest that the new tissue formed is fibrocartilage and the fate of the cells is unknown [8, 9]. Although MACI techniques also relieve pain, the long-term results using MACI are not yet available [10–12]. Similarly, the long-term clinical results for cartilage regeneration employing particulated juvenile allogeneic cartilage (DeNovo NT, Zimmer, Warsaw, IN) and the cartilage autograft implantation system (CAIS, Johnson & Johnson, New Brunswick, NJ) are not fully known [13–15]. In summary, the long-term outcomes of many cartilage restorative procedures are unsatisfactory and an improved method for joint surface repair is a clear unmet need in orthopaedic surgery.

Tissue engineering strategies combining chondrocytes or chondrocyte progenitor cells with biomimetic scaffolds made of natural or synthetic biomaterials could be a promising alternative for cartilage repair and regeneration. New cartilage matrix has been successfully produced in immunocompromised animals with cells placed on open fibrous scaffolds, such as collagen or polyesters, but these open lattice networks also permit invasion of inflammatory cells in immune competent animal models that can negatively affect matrix formation [16]. Hydrogels have high water content and can be modified to have diverse physical properties for use in medical implants, biosensors, and drug-delivery devices [17]. Prior to polymerization, hydrogels can be injected in a minimally invasive manner to fill defects of any size and shape [18–20]. Chondrocytes or chondroprogenitors can be homogenously suspended in a three-dimensional hydrogel, where the encapsulated cells can retain a rounded morphology that may induce or sustain a chondrocytic phenotype. The porous nature of hydrogels permits the transport of nutrients and waste. They also permit the translation of mechanical loads to encapsulated cells, similar to normal physiological conditions [21]. Although mechanically weak gels may be a drawback, the mechanical properties can be changed by crosslinking chemistry [22]. Besides, many degradable hydrogels degrade too fast to permit sufficient ECM production for neocartilage formation. Nonetheless, various research groups including ours have successfully demonstrated neocartilage tissue formation using multiple kinds of hydrogels to encapsulate chondrocytes or mesenchymal stem cells [23–28].

Poly(ethylene glycol) (PEG) is a linear carbon polymer that can be photochemically crosslinked into a stable hydrogel in which the biochemical and biophysical properties can be custom designed to obtain desirable properties that permit controlled cartilage matrix production. Our previous work has described a photocrosslinkable hydrogel for cell encapsulation using PEG polymer [21, 29]. PEG has a long history in medical applications with desirable chemistry that allows easy chemical modification. For example, the PEG polymer chain can be methacrylated and referred to as PEG diacrylate (PEGDM). In the presence of a photoinitiator (Igracure 2959, BASF Corp., Florham Park, NJ) and ultraviolet light (365 nm), the PEGDM forms crosslinks between the linear chains and forms a hydrogel. The polymers and gelation process allow easy placement into joint lesions and provide mechanical and structural stability during the regeneration process with desirable transport properties [30]. Previous work has shown that both the amount and distribution of extracellular matrix (ECM) by chondrocytes encapsulated in photopolymerized PEG hydrogels in vitro is directly correlated to the pore size, degradation rate, and swelling behavior of the gel networks [30–33]. When the network mesh pore size is too small (low swelling), the distribution of large ECM molecules (e.g. glycsosaminoglycans) is confined to the pericellular region. By increasing the pore size, the GAG molecules can diffuse throughout the intercellular spaces [32]. Additionally, the degradation properties of the PEG hydrogels (controlled by cross-linking density) can also affect the distribution of ECM. When chondrocytes are encapsulated in nondegradable PEG hydrogels, the collagen molecules are confined to the pericellular area, whereas the collagen is dispersed evenly into the void volume between the cells in gels that are designed to biodegrade [33]. The degradation characteristics can be tailored to make gels that degrade rapidly or slowly over time depending on the amount of degradable lactide units grafted to the PEG. Creating gels that have bimodal degradation could allow the scaffolds to retain their architecture during the tissue formation and remodeling processes.

Based on our earlier work on the degradation kinetics of copolymer gels, several candidate mixtures were tested in preliminary studies to formulate favorable PEGDM copolymer hydrogels that supported chondrocyte survival and growth, and allowed new matrix production [34]. We selected a degradable poly(ethylene glycol)-4,5 lactic acid dimethacrylate (PEG-LA-DM) and nondegradable PEGDM macromer for this study. Preliminary studies showed that PEG-LA-DM that was fully degradable dissolved too rapidly in vitro for the chondrocytes to make new ECM. Thus, the PEG-LA-DM was combined with nondegradable PEGDM in molar ratios that ranged from 90 % PEG-LA-DM/10 % PEGDM to a 50/50 ratio. The aims of our study were: 1) to demonstrate whether combining degradable and nondegradable PEGDM copolymer hydrogels would permit neocartilage formation by articular chondrocytes photoencapsulated into the gels; and 2) to investigate if the newly formed cartilage matrix could integrate with the native cartilage.

## Methods

The Institutional Animal Care and Use Committee of the Massachusetts General Hospital approved all procedures with animal tissues and live animals.

### Chondrocyte isolation

Swine articular cartilage was harvested from knee joints of three- to six- month-old female Yorkshire swine carcasses that were euthanized from other approved studies in our hospital (laboratory animal vendor: Tufts University, Grafton, MA). The cartilage was minced into 1 mm^3^ pieces and digested using 0.05 % collagenase type 2 (Worthington Biochemical Co., Freehold, NJ) solution for 16 h at 37 °C. After digestion the chondrocyte suspension was filtered through a 100 μm sterile cell strainer (Becton Dickinson, Franklin Lakes, NJ) to remove undigested debris. Cells were centrifuged and washed three times in phosphate-buffered saline. Using trypan blue and a hemacytometer, cell number and viability was assessed. Only cell isolations with viability above 90 % were used for the further experiments.

### Polymer preparation

The synthesis of degradable PEG-LA-DM and nondegradable PEGDM used in this study have been previously described [34]. Briefly, the macromer combinations were dissolved in sterile phosphate-buffered saline to a final concentration of 10 % (*w/w*). Mixed molar ratios of 50/50, 60/40, 70/30, 80/20 and 90/10 (PEG-LA-DM:PEGDM) were prepared for the study. 2-hydroxy-1[4-(hydroxyethoxy)phenyl]-2-methyl-1- propanone (Igracure; I2959) was used as the photosensitive initiator added to the polymer solutions at a final concentration of 0.05 % (*w/w*).

### Chondrocyte photoencapsulation

Swine articular chondrocytes were added to the macromer/photoinitiator solutions at a concentration of 6 × 10^7^ cells/mL. 100 μL of nonpolymerized gel containing the cells was placed in each cylindrical mould measuring 4.5 mm diameter × 6.5 mm in height. The gel-cell solutions were polymerized using ultraviolet (365 nm) irradiation at ~10 mW/cm^2^ for 10 min.

### Implantation and harvest

Experimental constructs were placed into subcutaneous pockets in 5-week-old athymic male mice (nu/nu) (MGH, Boston, MA) weighing 20–25 g and housed in a pathogen free facility. The animals were exposed to a 12 h light–dark cycle and allowed free access to sterile water and standard mouse chow. In a sterile biosafety cabinet the animals were anesthetized with an intraperitoneal injection of Avertin (250 mg/kg). A one centimeter incision was made through the skin on the dorsum of the mice and lateral subcutaneous pockets were made with blunt scissor dissection. The cell-scaffold implants were inserted into the pockets. The wound was infiltrated with 0.5 mL of sterile 0.5 % Bupivicaine for analgesia. At the time of specimen harvest the animals were euthanized by exposure to carbon dioxide from a gas source according to the recommendations of the American Veterinary Medical Association and death was confirmed by lack of breathing and heart beat.

A pilot study was performed in which implants from the five different polymer ratios (4/test polymer ratio) were made and implanted for 6 weeks. The results from the pilot study permitted us to focus only on the polymer ratios in which the matrix was contiguous and the construct volume was highest upon harvest. The full study focused only on ratios of 60/40 and 70/30. The sample size was 32 specimens for each polymer mixture. At 6, 12, and 18 weeks after implantation, eight specimens from each group were harvested. Specimens were evaluated for macroscopic appearance, weighed, and randomly selected for either histological examination or biochemical analyses (DNA; glycosaminoglycan (GAG); hydroxyproline). An additional 8 samples were prepared for each polymer mixture, but were not implanted for use as controls for assays at time zero.

### Ring model preparation

A previously published model using cartilage rings was used to study the integration of the engineered cartilage with existing cartilage matrix [35]. Using an 8 mm punch biopsy, disks of swine articular cartilage were made measuring 8 mm × 2 mm. Using a 5 mm biopsy punch, the center of the cartilage disk was removed leaving a ring of native cartilage matrix. To eliminate any influence on healing and integration by the chondrocytes in the rings, they were devitalized using five freeze–thaw cycles. Gel solution containing the chondrocytes cells was placed into the central cavities of the disks and photopolymerized. The ring model constructs were examined grossly and processed for histology to evaluate the interface between the native cartilage ring and the new cartilage formed in the copolymer gel.

### Specimen evaluation

Specimens in the pilot study were harvested at 6 weeks to assess the volume and cartilage forming ability among the various ratios of the copolymers. The dimensions and wet weight of the specimens were recorded. These specimens from the pilot study were only evaluated by histology. Samples were fixed in 10 % buffered formalin for a minimum of 24 h and embedded in paraffin. Serial 5 mm sections were made, deparaffinized, and stained with hematoxylin and eosin (H&E).

Specimens in the full study comparing 60/40 and 70/30 polymer ratios were collected after 6, 12, and 18 weeks in vivo. The specimens were cut in half with one-half used for biochemical assays and the remaining half for histological processing. In addition to H&E staining to evaluate overall tissue morphology, histological sections were stained with safranin O to assess proteoglycan content of the neotissue. Sections also were immunostained with antibodies against collagen types I and II (Chondrex, Redmond WA). Briefly, slides were treated with 2 % bovine testicular hyaluronidase at room temperature for 30 min. A blocking reagent consisting of 0.3 % hydrogen peroxide in methanol was added for another 30 min followed by 10 % goat serum for 30 min. On separate test slides, antibodies against collagen type I and type II were applied for 1 h. N-Universal Negative Control was applied for the negative control, and the secondary horseradish peroxidase (HRP) labelled antibody was added for 20 min. 3,3-diaminobenzidine was applied to each slide to react with the HRP, and the sections were counterstained with hematoxylin.

Samples of native articular swine cartilage and portions of the experimental samples were analyzed biochemically. The specimens were weighed to obtain the wet weight of the samples then lyophilized. After lyophilization, the dry weight of the specimens was recorded, and the difference between the wet and dry weights was the water content of the neotissue. Papain type III solution (Sigma-Aldrich, St. Louis, MO) at 125 mg/mL was added to the lyophilized specimens to digested the cartilage matrix for 16– 24 h at 60 °C. A PicoGreen dsDNA Quantitation Assay Kit (Molecular Probes, Eugene, Oregon) was used to determine the amount of DNA or cells in the specimens. A standard curve of Lambda DNA from the kit was generated. The amount of DNA from the test specimens was determined and used as an indicator of the proliferative potential of the photoencapsulated chondrocytes. A previously published dimethylmethylene blue dye method was employed to measure the amount of GAG in the specimens using chondroitin sulfate B (Sigma-Aldrich) as a standard in the interpretation of the data [36]. The amount of hydroxyproline in the tissues served as a surrogate marker for total collagen. Hydroxyproline was quantified using a previously published method using L-4- Hydroxyproline (Fluka Biochemika, Steinleim, Switzerland) as the standard [37]. All the DNA, GAG, and hydroxyproline data were normalized by wet tissue weight.

### Statistical analysis

Quantitative data of the study was statistically analyzed using a Student *t*-test (Sigmastat 2.0, SPSS Science, Chicago, IL) and ANOVA. All the values are reported as the mean ± standard deviation and the level of statistical significance was set at *p* < 0.05.

## Results

### Pilot study results

**Fig. 1:**
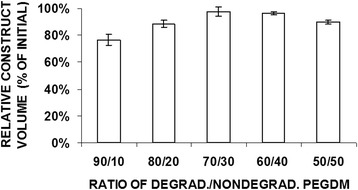
Construct volume data of the preliminary study after 6 weeks in vivo using 50/50, 60/40, 70/30, 80/20 and 90/10 ratios of degradable/nondegradable PEG (# *p* > 0.05)

**Fig. 2:**
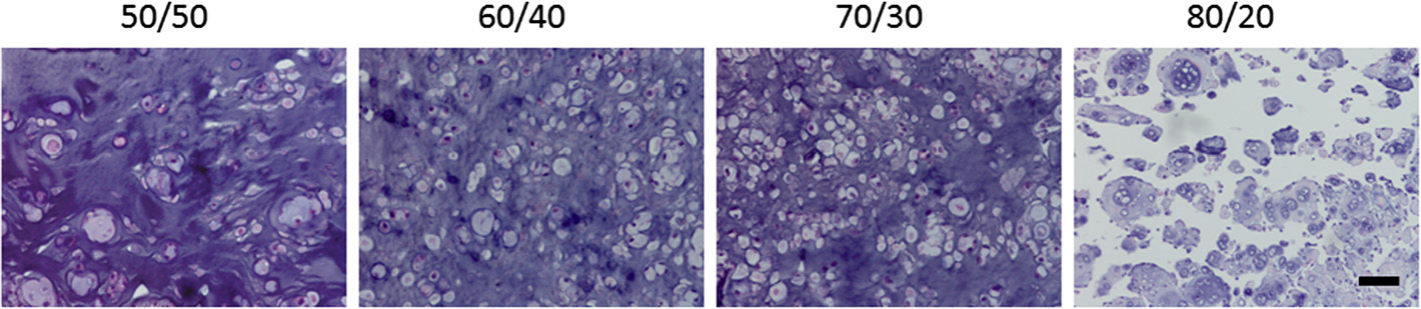
Hematoxylin and eosin-stained sections of the preliminary results demonstrated noncontiguous cartilage formation using 50/50, 60/40, 70/30, and 80/20 ratios. (From left to right, original magnification × 100, bar: 100 μm)

The volumes of the five different polymer ratios from the pilot study are shown in Fig. 1. Only specimens made with 60/40 and 70/30 retained greater than 90 % of the volume that was originally implanted. The histological results from the group made from 50/50 polymer showed isolated cells with small amounts of pericellular matrix surrounded by large areas of remaining PEG gel (Fig. 2). Specimens made from high amounts of degradable polymer, 80/20 and 90/10, had islands of cartilage, but large void spaces in between the cartilage areas. Specimens made with ratios of 60/40 and 70/30 demonstrated nearly contiguous cartilage matrix and were the focus of further study.

### Gross evaluation

The results for the full study are shown in Table 1. At 6 weeks there was a reduction in average wet weight in both copolymer hydrogel groups compared with the time zero control samples (*p* < 0.05 for 60/40 group and there’s no statistical difference for 70/30 group). However, the average wet weight of the specimens was higher than the initial weight in both groups by 18 weeks (*p* < 0.01). A reduction in average volume was observed at 6 weeks in 60/40 copolymer hydrogel group (*p* < 0.05), whereas there’s no significant reduction in volume in the 70/30 copolymer group at the same time point. Nonetheless, the volume of samples were higher than the initial volume in both groups (*p* < 0.01) by 18 weeks. There was a very slight reduction in average water content at 6 weeks in both copolymer groups compared with the native cartilage control samples, and by 18 weeks, there’s slight increase in average water content in both groups. However, there were no statistical differences in water content among the study groups or between the different harvest times.

**Table 1:** Summary of Specimen Data^a^

	60/40 (PEG-LA-DM/PEGDM)				70/30 (PEG-LA-DM/PEGDM)			
	Time 0 Control^b^	6 weeks	12 weeks	18 weeks	Time 0 Control	6 weeks	12 weeks	18 weeks
Wet weight (mg)	103.50 ± 4.10	91.35 ± 4.31	111.05 ± 4.59	117.90 ± 4.38	104.80 ± 8.06	100.35 ± 3.61	107.45 ± 2.57	114.30 ± 2.54
Volume (mm^3^)	93.54 ± 1.21	86.09 ± 3.92	96.02 ± 3.89	104.55 ± 1.32	92.92 ± 4.64	91.87 ± 1.42	93.79 ± 1.38	102.85 ± 2.37
Water content (%)	82.29 ± 3.78	79.01 ± 4.05	85.70 ± 2.45	86.09 ± 2.95	83.14 ± 3.87	80.07 ± 4.42	86.22 ± 3.17	85.61 ± 2.91

*PEG-LA-DM* poly(ethylene glycol)-4,5 lactic acid dimethacrylate, *GAG* glycosaminoglycan, *HYP* hydroxyproline

^a^All the data are presented as mean value ± standard deviation

^b^Time 0 controls were specimens in which cells were encapsulated in gels and then analyzed for baseline data

**Fig. 3:**
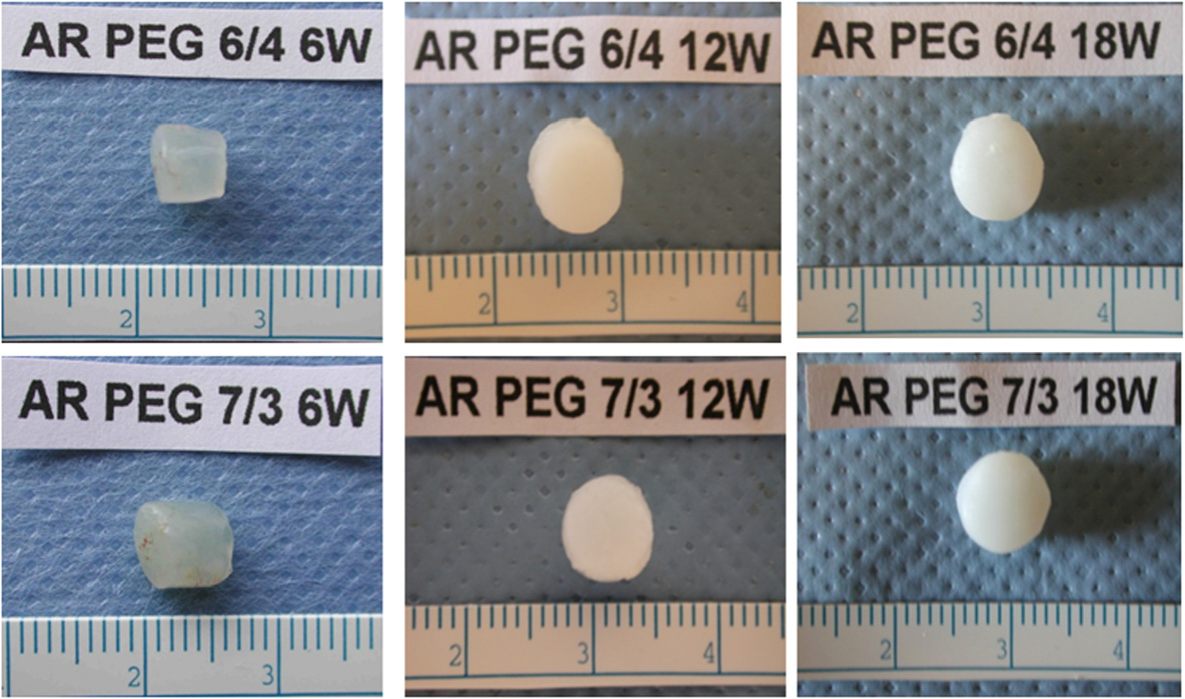
Macroscopic view of constructs over the implantation time for 60/40 and 70/30 ratios

At the early 6-week time point the samples remained translucent due to the residual gel component. The 6-week samples also were relatively soft and gel-like. As the gel was replaced with new cartilage matrix over time, the specimens became increasingly opaque (Fig. 3).

### Biochemical evaluation

**Fig. 4:**
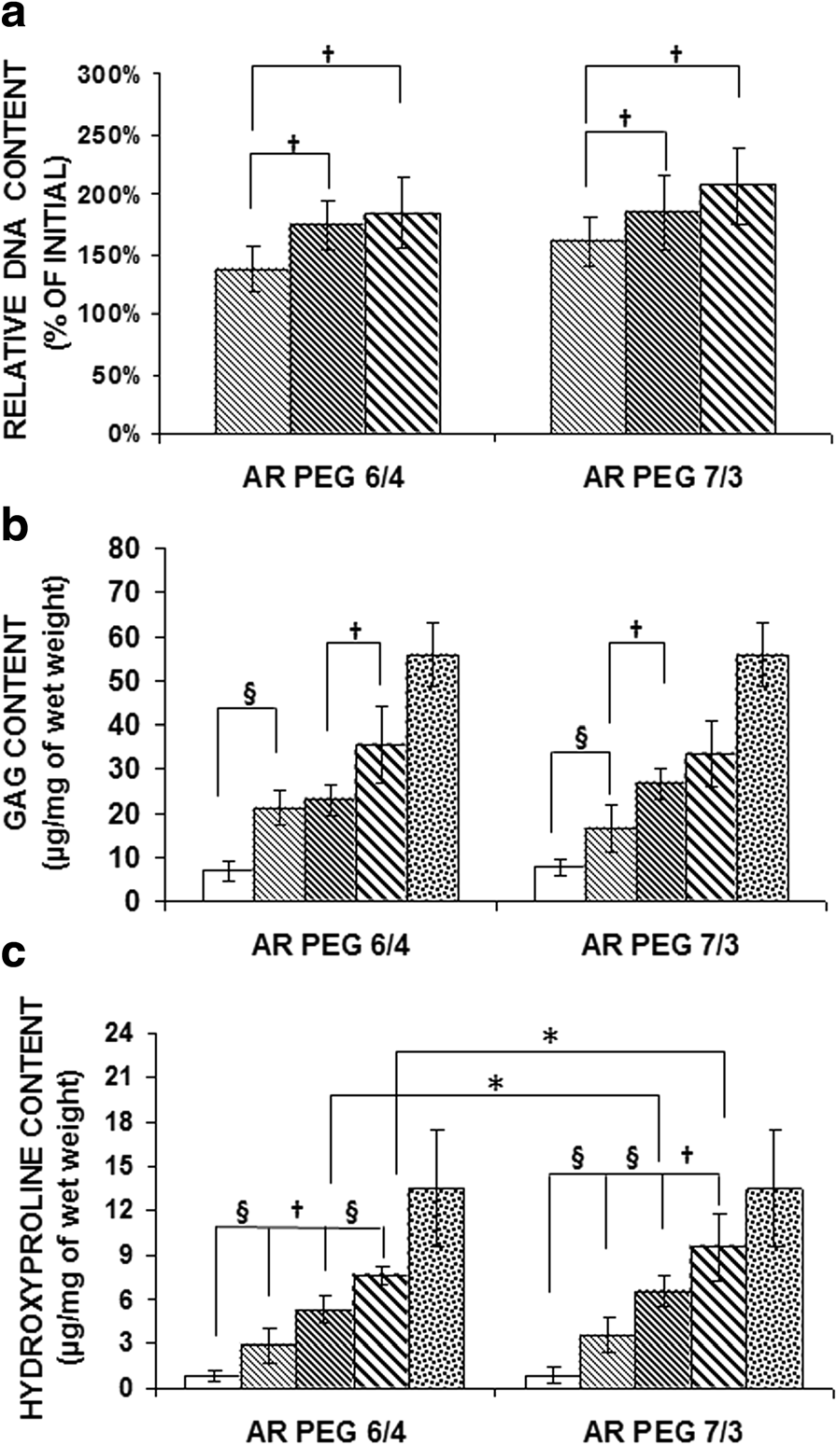
Biochemical evaluation data. (**a**) DNA content (**b**) GAG content and (**c**) hydroxyproline content. ( 0w,  6w,  12w,  18w,  native swine) (* *p* < 0.05, † *p* < 0.01, § *p* < 0.001)

The DNA content of copolymer/cell constructs at time zero was measured and set as 100 % for comparision of the experimenatl implants (Fig. 4a) Specimens from both copolymer groups increased over time suggesting that the cells were proliferating within the gels. By 18 weeks, the average amount of DNA in the specimens was 184.29 ± 29.19 and 206.74 ± 32.62 for both 60/40 and 70/30 hydrogel groups, respectively. There were no significant differences noted among the groups over the time points studied.

The amount of GAG in the constructs increased over time for both copolymer groups (Fig. 4b). By 18 weeks, the amount of GAG in the 60/40 group was 35.32 ± 8.67 μg/mg of wet tissue weight, whereas it was slightly lower in the 70/30 group, 33.46 ± 7.32 μg/mg. There was no statistically significant difference between different study groups at each of the harvest time points. The amount of GAG in the engineered cartilage made with 60/40 group was 63.39 % of that in native articular cartilage at the 18-week time point, whereas the amount of GAG in the 70/30 at this timegroup was 60.06 % of that in native articular cartilage.

The amount of hydroxyproline, used as a surrogate measure of total collagen, also increased over time. The amount of toal collagen at 18 weeks in the 60/40 group was 7.61 ± 0.60 μg/mg of wet tissue weight compared to 13.47 ± 3.95 μg/mg in native articular cartilage. Stated differently, this was about about 56.52 % of that found in the native cartilage. In both groups, the hydroxyproline content (Fig. 4c) also increased over time. At the final harvest time point, the total collagen in the 60/40 hydrogel group 7.61 ± 0.60 μg/mg of wet tissue weight compared to 13.47 ± 3.95 μg/mg of wet tissue weight in native articular cartilage, or about 56.52 % of that found in the native cartilage. The total collagen was slightly higher the 70/30 hydrogel group measuring 9.52 ± 3.01 μg/mg or about 70.67 % of the content measured in native cartilage. Both groups only reached about one-half to two-thirds of the amount of collagen on native articular cartilage over the time period studied. There was no significant difference between groups at 18 weeks, however.

### Histological evaluation

**Fig. 5:**
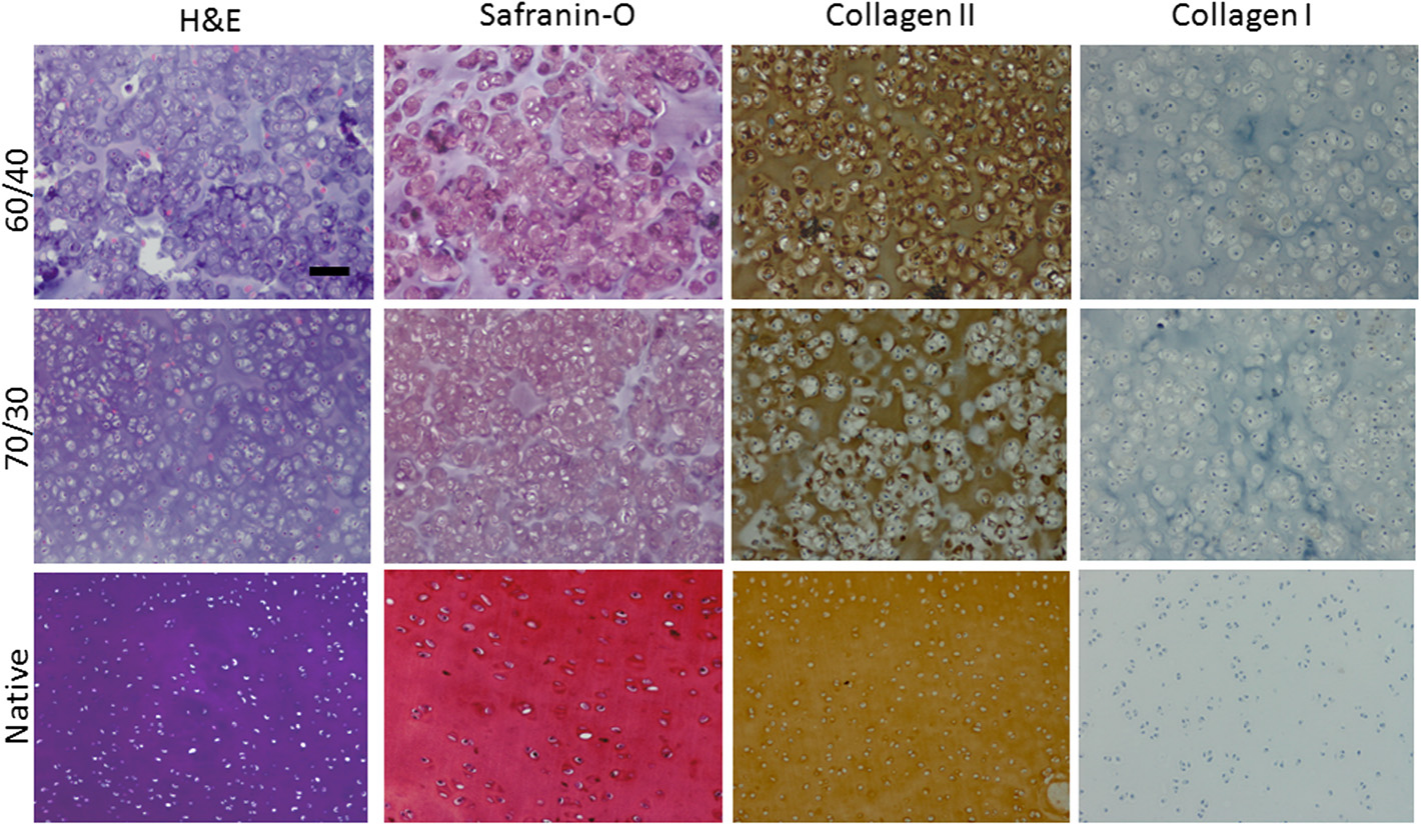
Histological and an immunohistochemical results from gels made with 60/40 and 70/30 ratios of degradable/nondegradable PEG compared to native swine articular cartilage. (Original magnification × 100, bar: 100 μm)

Typical of the morphology found in native articular cartilage, chondrocytes were observed in lacunae surrounded by basophilic extracellular cartilage matrix in histological specimens from the engineered constructs (Fig. 5). The specimens were hypercellular and the number of cells nearly doubled over time of the study (Fig. 4a). The distribution of the encapsulated chondrocytes at 6 weeks was nonhomogeneous in both study groups and the cells appear to be somewhat hypertrophic. This may be due to the incomplete degradation of the hydrolysable crosslinks of the polymer scaffold at the early time point. Over time, the new cartilage matrix became more homogeneous and there were no discernible visual differences between the 60/40 and 70/30 groups by 18 weeks.

The results from immunohistochemistry demonstrated that the cells encapsulated within the photopolymerized gels could maintain their chondrocyte phenotype by producing collagen type II, typical of that found in native cartilage (Fig. 5). At 6-weeks, the collagen type II staining was restricted to the pericellular regions of the encapsulated cells, probably because of residual polymer at this time point (data not shown). Over time, the type II collagen staining pattern became more homogeneous in the engineered specimens, similar to the observation in native swine cartilage (Fig. 5). Little evidence of type I collagen was noted in the specimens.

**Fig. 6:**
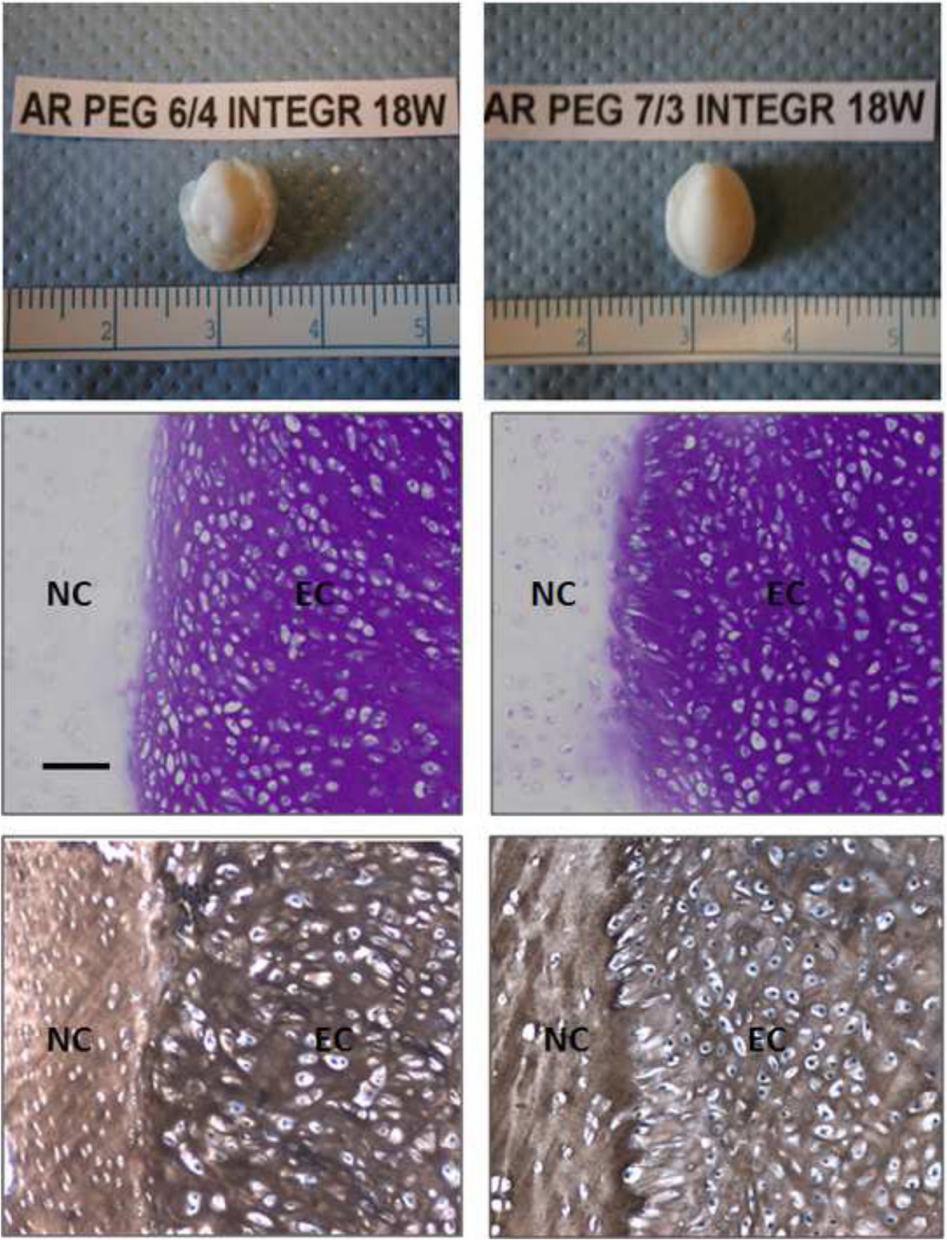
Upper row: Macroscopic view of 18 week constructs of ring model for integration study. Below each group are the toluidine blue (*left*) and collagen type II (*right*) staining of the integration interface between the engineered cartilage (EC) and native articular cartilage (NC) related to the above constructs. (Original magnification × 100, bar: 100 μm)

The neocartilage generated in the colpolymers was capable of integrating with native cartilage as demonstrated in the cartilage ring model. Devitalized cartilage rings were used to eliminate the potential for influence by the cells that reside in the native cartilage. Histological sections at the interface of the engineered cartilage and native cartilage showed integration between the tissues (Fig. 6). The cells at the interface were ovoid and perpendicular to the surface of the native cartilage. The engineered tissue filled irregularities of various depths and shape along the interface.

## Discussion

Development of effective and minimally invasive tissue engineering techniques using injectable hydrogel materials to support chondrocyte growth and ECM production could be an ideal therapeutic strategy for cartilage repair and regeneration. Numerous hydrogels have been formulated for cartilage generation including natural materials such as fibrin, chitosan, collagen, hyaluronic acid (HA), and self-assembling peptides to mention a few [19, 27, 38–40]. Synthetic materials also have been developed into hydrogels that permit encapsulation of chondrocytes and new cartilage matrix formation, such as poly(vinyl alcohol) and poly(ethylene glycol) [18, 31]. Extracellular matrix components, such as GAG or HA, or growth factors have been added to these gels to promote cartilage formation in some cases [41]. Most studies, however, have explored only single, base polymer formulations and not combinations thereof.

Gels can be formulated that are non-degradable and resistant to breakdown allowing maintenance of three-dimensional architecture. However, these resilient gels inhibit cell-to-cell contact and interfere with intracellular ECM production. Data from previous studies show that nondegradable PEGDM gels permit extracellular matrix formation in the pericellular region, but the nondegradable polymer interferes with generating contiguous cartilage matrix [32]. Synthesizing degradable PEG gels that can break down within predictable periods of time can be achieved by adding in water soluble crosslinks to the macromer backbone. It is challenging, however, to delicately time the degradation process while maintaining volume and three-dimensional shape in vivo. Photochemical crosslinking to polymerize gels allows for molding them into predetermined shapes permitting cartilage formation in the desired form. Previously published in vitro data showed that fully degradable photocrosslinked PEG-LA-DM degraded too rapidly for cartilage matrix to form [34]. Pilot studies in vivo with this degradable formulation confirmed this as well. By combining nondegradable with degradable PEG, we hoped to tailor the gels to form articular cartilage yet maintain structural integrity during tissue formation. Thus, the goal of this study was to evaluate neocartilage formation employing copolymers composed of a combination of degradable and nondegradable PEG macromers.

Modifications of the polymerization process can affect the mechanical and biological behaviors of the final hydrogel. The amount of crosslinking has a direct impact on the cartilage formation capacity of the chondrocytes [21], although the crosslinking process does not negatively affect cell viability throughout the gels [30]. Also, Omobono et al. showed that secondary treatment of photochemically crosslinked collagen gels with chemical crosslinking using N-hydroxysuccinimide (NHS) and 1-ethyl-3-(3-dimethylaminopropyl)carbodiimide (EDC) was able to slow the enzymatic degradation without compromising cell viability [42]. In the current study, we chose two polymer formulations that could be photopolymerized simultaneously with the same photoinitiator agent and the same wavelength of light. This unique strategy permitted us to blend these two different PEG formulations with the cells and polymerize them simultaneously into the final hydrogel promoting cartilage matrix formation. The pilot study confirmed that gels high in degradable PEG (80/20 and 90/10) content dissolved too rapidly for cohesive neocartilage formation. Formulations where the nondegradable PEG content was 50 % inhibited contiguous cartilage matrix formation due to the resistance of the polymer to biodegradation. These results narrowed the focus of the larger study on the photochemically crosslinked co-polymer formulations of 60/40 and 70/30.

Our data showed that the average weight and volume of the neocartilage samples were statistically higher than the initial values at the time of implantation for both groups (60/40 and 70/30) at 18 weeks. The neocartilage samples were increasingly opaque over time indicating more ECM deposition. The changes in the biochemistry of the ECM paralleled the changes in morphology over time. There was a significant increase in GAG, hydroxyproline, and total DNA content during the 18 weeks of study, and there were no statistical differences between the 60/40 and 70/30 groups at each time point. Normally, articular chondrocytes in cartilage are nurished by diffusion from the surrounding synovial fluid facilitated by the loading patterns of the joint. Ectopic implantation of these cells into subcutaneous space alters dramatically their biological environment, while the nutritive elements received from the interstitial fluid and the biomechanical stimuli of the joint are quite different.

The strength of the interface of the engineered cartilage with existing adjacent native cartilage is of great importance in articular cartilage repair because weakness at this interface could cause collapse of the new cartilage tissue and jeopardize the cartilage restoration. Promoting integration of the new and existing matrix is correlated with collagen synthesis and deposition by viable cells at the interface [43–46]. The results from this study showed that the engineered cartilage integrated with existing native cartilage using these copolymer hydrogel formulations seeded with cells. Partial integration of the neocartilage with the opposing surface of the native cartilage was noted by 6 weeks (data not shown), but by week 12 and 18, the integration interface showed repopulation, deposition of matrix macromolecules, and tight adhesion between the the engineered and native cartilage. The improved integration and the tight interface junction at the later times may be related to the breakdown of the polymer scaffold over time with the simultaneous growth of new cartilage matrix.

Most studies evaluating polymer scaffolds seeded with cells have been initially tested in vitro prior to pilot testing by implantation into nude mice. Nude mice lack a repertoire of mature T-cells and cannot mount a cellular rejection response to allogeneic or xenogeneic cells. In many ways they can be considered a “living” petri dish for studying cartilage (and bone) matrix formation from cell-seeded scaffolds. We chose a mouse model with subcutaneous implantation to generate neocartilage because this permits better cartilage formation than in vitro systems. The biomechanical microenvironment in the joint cannot be replicated in the subcutaneous pockets on the back of nude mice, however. We recognize that the model may have some deficiencies, but the nude mouse is a suitable small animal model to pilot screen and test new scaffolds and tissue formation. Further modifications in hydrogel chemistry or photopolymerization mechanism can improve the scaffold properties of PEG derivatives for tissue engineering of articular cartilage, particularly in terms of the intrinsic adhesive properties. The application of these copolymers in immunocompetent animal models will be the focus of subsequent studies.

## Conclusions

Our findings demonstrated that copolymers that are composed of degradable and nondegradable PEGDM macromers are favorable scaffolds for engineering articular cartilage. No obvious differences were observed among the studied copolymers at different ratios (60/40 and 70/30) in the cell proliferation, biochemical performance and integrative properties between the engineered tissue and adjacent native tissue. Potentially, a liquid macromer/chondrocyte suspension like PEGDM copolymer could be injected into the cartilaginous defect and photopolymerized to provide a minimally invasive technique to promote or enhance articular cartilage repair. Further studies are required to investigate the potential of this tissue engineering approach for chondrogenesis involving large animal models and focusing on the biomechanical properties of the engineered articular cartilage.

### Abbreviations

PEG, poly (ethylene glycol); PEGDM, poly (ethylene glycol) dimethacrylate; ECM, extracellular matrix; ACI, autologous cell implantation; OATS, osteoarticular autograft transfer system; H&E, hematoxylin and eosin; GAG, glycosaminoglycan; HYP, hydroxyproline; ANOVA, Analysis of variance
